# Water influx into cerebrospinal fluid is significantly reduced in senile plaque bearing transgenic mice, supporting beta-amyloid clearance hypothesis of Alzheimer’s disease

**DOI:** 10.1179/1743132814Y.0000000434

**Published:** 2014-12

**Authors:** Hironaka Igarashi, Yuji Suzuki, Ingrid L Kwee, Tsutomu Nakada

**Affiliations:** 1 Center for Integrated Human Brain Science, Brain Research Institute, University of Niigata, Chuo-ku, Niigata, Japan; 2 Department of Neurology, University of California, Davis, Martinez, CA, USA

**Keywords:** MRI, JJVCPE, Aquaporin, Virchow–Robin space, Interstitial flow

## Abstract

Recent studies on cerebrospinal fluid (CSF) homeostasis emphasize the importance of water influx into the peri-capillary (Virchow–Robin) space through aquaporin 4 (AQP-4). This water flow is believed to have the functionality equivalent to the systemic lymphatic system and plays a critical role in beta-amyloid clearance. Using a newly developed molecular imaging technique capable of tracing water molecules, *in vivo*, water influx into the CSF was quantitatively analyzed in senile plaque (SP) bearing transgenic Alzheimer’s disease (AD) model mice. The results unequivocally demonstrated that water influx into CSF is significantly impaired in SP-bearing transgenic mice, the degree of which being virtually identical to that previously observed in AQP-4 knockout mice. The study strongly indicates that disturbance in AQP-4-based water flow and, hence, impairment in beta-amyloid clearance play a significant role in SP formation.

## Abbreviations

CSF, cerebrospinal fluid; AQP-4, aquaporin 4; AD, Alzheimer’s disease; MRI, magnetic resonance imaging; PET, positron emission tomography; JJVCPE, JJ vicinal coupling proton exchange; SP, senile plaque; FAD, Familial Alzheimer's Disease; APP, amyloid precursor protein; PS1, Presenilin-1; WT, wild type; SpO_2_, oxygen saturation; RARE, rapid acquisition with refocused echoes; NMR, nuclear magnetic resonance; AQP-1, aquaporin 1; BBB, blood brain barrier; ROI, region of interest; BG, basal ganglia.

## Introduction

The classic model of CSF homeostasis calls for the circulation theory where the majority of CSF is thought to be produced by the choroid plexus and circulates from the ventricles into the subarachnoid spaces. Recently, however, this circulation model has come under serious challenge. It is now believed that CSF is produced and absorbed throughout the entire CSF system, and the peri-capillary (Virchow–Robin) space plays a critical role in CSF production.^1^ CSF flow through the Virchow–Robin space, which has long been known as “interstitial flow” and believed to play a role equivalent to systemic lymphatic, has recently re-gained substantial attention due to its relation to beta-amyloid clearance.^2^^–^4

Using a newly developed *non-invasive* imaging method capable of tracing exogenously applied substrates by magnetic resonance imaging (MRI) equivalent to positron emission tomography (PET), JJ vicinal coupling proton exchange (JJVCPE) imaging,^5^,^6^ we successfully demonstrated that water flow into the CSF system is regulated by aquaporin-4 (AQP-4), the water channel abundantly present on perivascular end feet of astrocytes.^7^ In this study, we demonstrated that this water flow is significantly reduced in senile plaque (SP) bearing transgenic Alzheimer disease (AD) model mice.

## Materials and Methods

### Animal preparation

The study was approved by the Internal Review Board of University of Niigata and carried out in accordance with the Guidelines laid down by the NIH in the US regarding the care and use of animals for experimental procedures.

Male B6SJL-Tg (APPSwFlLon, PSEN1*M146L*L286V) 6799Vas/Mmjax mice (APP-PS1 Tg mice, 1–2 months of age) were obtained from Jackson Laboratory (Bar Harbor, Maine, USA), and raised in our laboratory until 18 months of age. These transgenic mice overexpress both mutant human amyloid precursor protein (APP) (695) with the Swedish (K670N, M671L), Florida (I716V), and London (V717I) Familial AD (FAD) mutations and human Presenilin-1 (PS1) harboring two FAD mutations, M146L and L286V. Expression of both transgenes is regulated by neural-specific elements of the mouse Thy1 promoter to drive overexpression in the brain.^8^ Animals were housed in standard housing conditions with a 12-hour light/dark cycle and were provided with water and food *ad libitum*.

APP-PS1 Tg mice and wild-type (WT) mice (both sexes, 7–8 months old), breathing spontaneously and anesthetized with an intra-peritoneal administration of urethane (1.2 g/kg), were positioned on their backs in a custom-made Plexiglas stereotactic holder. Their head was fixed in position by ear and tooth bars. Rectal temperature was maintained at 37 ± 0.5°C using a custom-designed temperature control system. Oxygen saturation (SpO_2_) was monitored throughout the MR study using a pulse oxymeter Mouse Ox (STARR Life Sciences Co, Oakmont, PA, USA) with probe placement on the left thigh. Data from animals showing SpO_2_ of less than 93% of SpO_2_ at any point in the experiment were discarded (one WT mouse). Five mice of each group accomplished completed the experiment. Normal saline, 0.2 ml, containing 20% of H_2_^17^O was administered as an intravenous bolus injection at the 75th phase (10 minutes after the first scan) using an automatic injector at 0.04 ml/second through PE10 tubing inserted into the right femoral vein.

### Imaging parameters

MRI experiments were performed on a 15 cm bore 7 T horizontal magnet (Magnex Scientific, Abingdon, UK) with a Varian Unity-INOVA-300 system (Varian Inc., Palo Alto, CA, USA) equipped with an actively shielded gradient. A custom made one turn surface coil, 20 mm of outer diameter, was used for RF transmission. Adiabatic double-spin echo-prepared rapid acquisition with refocused echoes (RARE) was utilized with the following parameter settings: single slice (2 mm thick), 128 × 128 matrix image of 20 × 20 mm field of view for every 8 seconds, TR of 2000 milliseconds, Echo Train of 32, TE for first echo of 8.8 milliseconds, echo spacing of 5 milliseconds, and effective TE of 84.8 milliseconds. Imaging slabs were set 6 mm caudal from the top of the cerebrum. A total of 525 phases (scan time 70 minutes) were obtained at 8-second intervals.

### Data analysis

**Figure 1:**
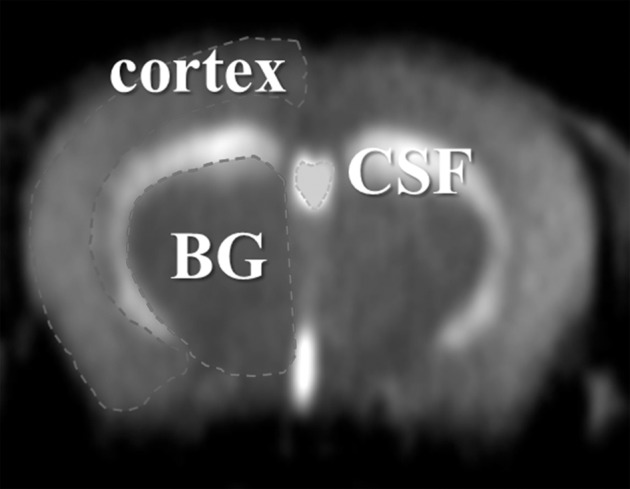
Regions of interests (ROIs) were selected semi-automatically using image processing software. BG: basal ganglia, CSF: cerebrospinal fluid.

**Figure 2:**
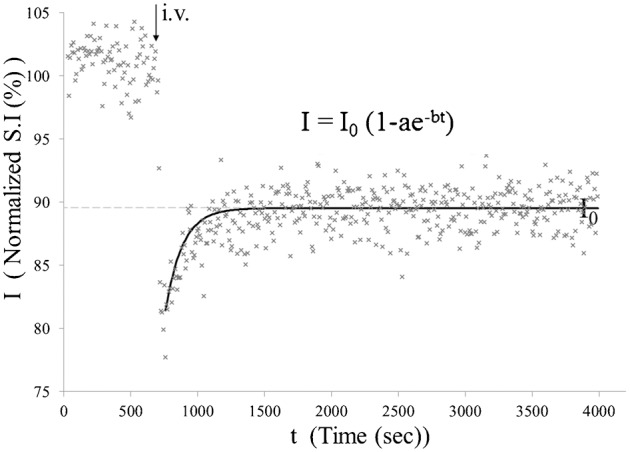
Decay curve fitting. Intensities at the steady state of each area, expressed as% against the averaged intensity of identical pixel prior to administration of H_2_^17^O, were determined by fitting their time course using the function: *I*  =  *I*_0_(1 − *ae*^−*bt*^). *I*_0_ denotes the normalized signal intensity at infinite time (*t*  =  ∞) calculated from the fitted curve.

Images were analyzed by image processing software (MR vision; MRVision Co. Winchester, MA, USA). Averaged% intensities, which reflect relative influx of H_2_^17^O in three areas (Fig. 1) namely cortex, basal ganglia (BG), and third ventricle, were plotted against time. Intensities at the steady state of each area, expressed as% against the averaged intensity of identical pixel prior to administration of H_2_^17^O, were determined by fitting their time course by the function (Fig. 2):
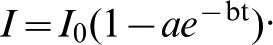
Subsequently, numerical data were subjected to ANOVA with Fisher’s Partial Least-Squares Differences (PLSD) *post hoc* test for group analysis. The value of *P* < 0.05 was regarded as statistically significant. All data are shown as mean ± standard deviation.

### Immunohistochemistry

Brain tissues were taken from the anesthetized mice immediately after the MRI experiments and fixed in 4% paraformaldehyde. The tissues were dehydrated with ethanol and embedded in paraffin wax. Serial sections (10 μm thickness) were cut from the paraffin block and immunostained using mouse monoclonal antibodies against amyloid-beta (IBL, Tokyo, Japan, 1 : 50). Sections were pre-treated with formic acid and immunolabeling was detected using the avidin–biotin–peroxidase complex method with Vectastain ABC kit (Vector, Burlingame, CA, USA), and visualized with diaminobenzidine/H_2_O_2_ solution. Counterstaining was carried out with hematoxylin. Specimens were observed using light microscopy (AX80T; Olympus, Tokyo, Japan) and images were captured using a digital camera (DP71; Olympus).

## Results

**Figure 3:**
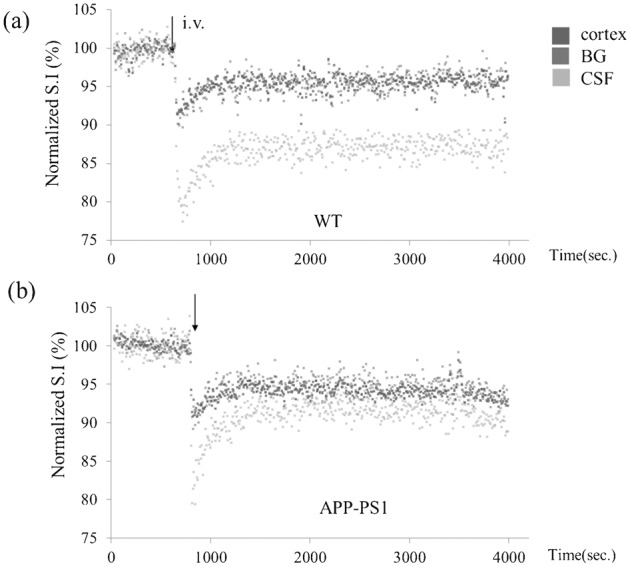
Representative time course. Representative time course of signal intensities within pixels of each region of interest (ROI) shown in Fig. 1 following intravenous H_2_^17^O administration in transgenic (APP-PS1) and age-matched wild-type (WT) mice. Blue: cortex, red: basal ganglia (BG), green: cerebrospinal fluid (CSF) within the third ventricle. Each dot represents the intensity of each pixel within the ROI.

**Figure 4:**
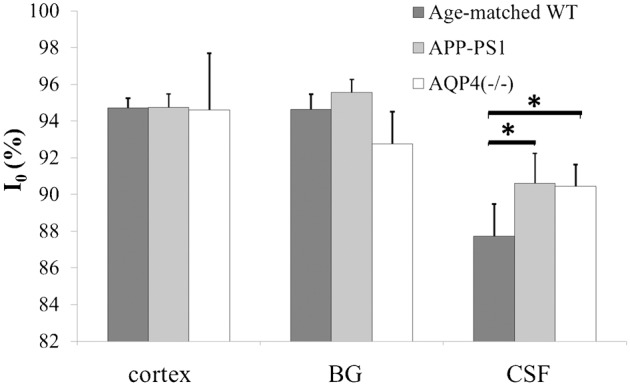
*I*_0_ of three ROIs. Values of *I*_0_ in cortex and basal ganglia (BG) are virtually identical between APP-PS1 and WT mice. In contrast, *I*_0_ of CSF within the third ventricle is significantly higher in APP-PS1 mice. For comparison, previously reported values of aquaporin-4 (AQP-4) knock out mice are shown together. *P < 0.05. WT, wild type; APP-PS1, APP-PS1 transgenic; AQP4(−/−), AQP4 knockout.

**Figure 5:**
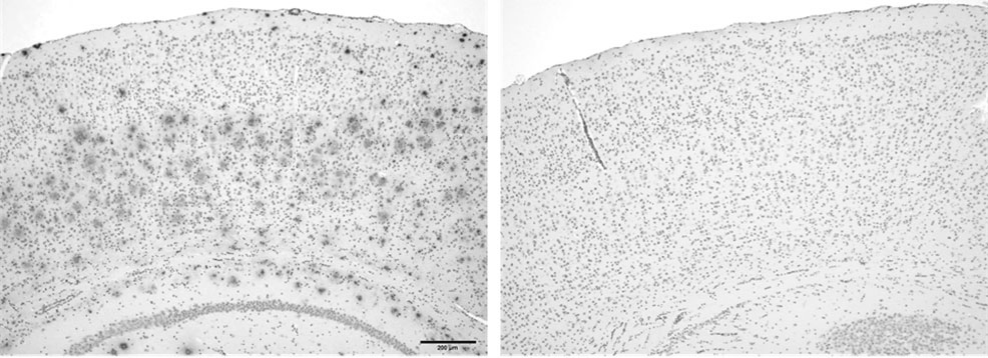
Beta-amyloid immunohistology of APP-PS1 transgenic (left) and control (right) mice.

Time course of signal intensity changes after intravenous injection of 20% of H_2_^17^O for cortex (blue), BG (red), and third ventricle (green) in a representative transgenic (APP-PS1) and age-matched WT mouse are shown in Fig. 3. It is noted that water penetrated into the brain very rapidly and the concentration of H_2_^17^O plateaued within 20 minutes. In WT, penetration and subsequent steady concentration of H_2_^17^O is significantly higher within the third ventricle compared to brain parenchyma (cortex and BG). In contrast, penetration and steady concentration of H_2_^17^O in the third ventricle is significantly reduced in APP-PS1 mice. Group analysis statistically confirmed these findings (Figure 4). Previously reported data on AQP-4 knock out mice are added for comparison.^7^ SP harboring in APP-PS1 mice was confirmed with immunohistochemistry (Fig. 5).

## Discussion

Molecular imaging using MRI was originally introduced as imaging studies of endogenous molecules such as lactate. Subsequently, tracer technique for similar nuclear medicine was developed utilizing exogenous ligands containing 19-fluorine.^9^–^11^ Attempts to develop ligand-based molecular MRI akin to PET imaging, however, were hampered primarily due to incompatibility between potential probe (charged molecules such as gadolinium) and membrane permeability. This fundamental problem has long stymied effective development of ligand-based molecular imaging in the field of MRI. Recently, we successfully developed a versatile technique capable of tracing an exogenously applied ligand with MRI using a non-radioactive, nuclear magnetic resonance (NMR) sensitive oxygen isotope, O^17^. The technique is referred to as JJVCPE imaging.^5^,^6^,^8^ This technique, as demonstrated in this study, allows for the effective tracing of water molecules, *in vivo*, in the form of imaging.

There are no conventional lymphatics within the brain. Physiological studies have long been suggested that CSF circulation is the brain’s equivalent of the lymphatic system. There is increasing evidence indicating that extracranial lymphatic vessels play a main role in CSF drainage from the brain. CSF carries solutes from brain parenchyma along peri-capillary spaces. Tracers injected into gray matter drain along basement membranes in the walls of capillaries and arteries (interstitial flow). Drainage of antigens from the brain by this route may play a significant role in the immune response in virus infections, experimental auto-immune encephalomyelitis and multiple sclerosis as well as beta-amyloid clearance.^3^,^4^ This study clearly demonstrated that SP-bearing transgenic AD model mice exhibit significant reduction of water influx into the CSF system. Although it is difficult to judge whether it is the primary cause or a secondary result, dysfunction in the AQP-4-based “lymphatic” system of the brain is significantly correlated with SP formation.

The aquaporin family is a large collection of integral membrane proteins that enable the movement of water across biological membranes.^12^,^13^ While aquaporin 1 (AQP-1) is abundantly expressed in systemic capillaries, AQP-1 is actively suppressed in brain capillaries.^14^ This is believed to be an essential property of the brain for proper maintenance of the blood–brain barrier (BBB), preventing excessive movement of water across capillary walls. Control of water drainage into the systemic lymphatics is also AQP-1 dependent and, in general, considered to be much more loosely regulated than those of capillaries. Should the brain need to suppress AQP-1 expression for proper BBB functionality, a total lack of conventional lymphatics appears desirable. Since the main functionality of the BBB is believed to reside within the selectivity of the tight junctions between endothelial cells that restricts the passage of solutes and AQP-4 on perivascular end feet of astrocytes connects the intracellular space of astrocytes and peri-capillary space,^7^,^15^ the CSF system is still within the BBB. Drainage of debris into the systemic lymphatic system undergoes molecular scrutiny before entering venous circulation. Therefore, it is highly conceivable that drainage of debris into the CSF through Virchow–Robin space flow such as in the case of beta-amyloid would be subjected to similar molecular scrutiny. In this matter, the ventricular system plays a role similar to lymph nodes of the systemic lymphatic system in neutralizing toxicity of some proteins. Indeed, prealbumin (transthyretin), the protein abundantly present in the CSF, is found to be a chaperon for beta-amyloid, and prevents beta-amyloid’s natural tendency to accumulate into plaques.^16^

## Conclusion

In conclusion, our study clearly demonstrated the significant reduction in water influx into the CSF in SP-bearing transgenic AD model mice. The finding strongly supports that impairment in clearance of beta-amyloid may likely be the necessary factor in the pathophysiology of SP formation. Cohort studies for assessing dynamic indices of the balance between production and clearance of beta-amyloid in clinical populations will be critical for our proper understanding of the pathogenesis of AD.

## Disclaimer Statements

**Contributors** There are no contributors except the four authors.

**Funding** Japan Society for the Promotion of Science.

**Conflicts of interest** There is no conflict-of-interest.

**Ethics approval** The study was approved by the Internal Review Board of University of Niigata and carried out in accordance with the Guidelines laid down by the NIH in the US regarding the care and use of animals for experimental procedures.

## References

[1] Orešković D, Klarica M (2010). The formation of cerebrospinal fluid: nearly a hundred years of interpretations and misinterpretations.. Brain Res Rev..

[2] Johnston M, Papaiconomou C (2002). Cerebrospinal fluid transport: a lymphatic perspective.. News Physiol Sci..

[3] Weller RO, Djuanda E, Yow HY, Carare RO (2009). Lymphatic drainage of the brain and the pathophysiology of neurological disease.. Acta Neuropathol..

[4] Iliff JJ, Wang M, Liao Y, Plogg BA, Peng W, Gundersen GA (2012). A paravascular pathway facilitates CSF flow through the brain parenchyma and the clearance of interstitial solutes, including amyloid β.. Sci Transl Med..

[5] Nakada T (2007). Grant-in-aid for scientific research (S). Integrated science and innovative science.. Magnetic Resonance Molecular Microimaging.

[6] Suzuki K, Igarashi H, Huber VJ, Kitaura H, Kwee IL, Nakada T (2014). Ligand based molecular MRI: O-17 JJVCPE amyloid imaging in transgenic mice.. J Neuroimaging.

[7] Igarashi H, Tsujita M, Kwee IL, Nakada T (2014). Water influx into cerebrospinal fluid (CSF) is primarily controlled by aquaporin-4, not by aquaporin-1: O-17 JJVCPE MRI study in knockout mice.. NeuroReport..

[8] Oakley H, Cole SL, Logan S, Maus E, Shao P, Craft J (2006). Intraneuronal beta-amyloid aggregates, neurodegeneration, and neuron loss in transgenic mice with five familial Alzheimer's disease mutations: potential factors in amyloid plaque formation.. J Neurosci..

[9] Nakada T, Kwee IL (1987). Heterogeneity of regional cerebral glucose metabolism demonstrated in situ by 19F FDG NMR rotating frame zeugmatography: one dimensional chemical shift imaging of normal and gliosarcoma implanted brain.. Magn Reson Imaging..

[10] Nakada T, Kwee IL, Card PJ, Matwiyoff NA, Griffey BV, Griffey RH (1988). 19-Fluorine NMR imaging of glucose metabolism.. Magn Reson Med..

[11] Nakada T, Kwee IL, Griffey BV, Griffey RH (1988). 19F NMR glucose metabolic imaging in rabbit.. Radiology..

[12] Nielsen S, Smith BL, Christensen EI, Agre P (1993). Distribution of the aquaporin CHIP in secretory and resorptive epithelia and capillary endothelia.. Proc Natl Acad Sci USA..

[13] Huber VJ, Tsujita M, Nakada MT (2012). Aquaporins in drug discovery and pharmacotherapy.. Mol Aspects Med..

[14] Dolman D, Drndarski S, Abbott NJ, Rattray M (2005). Induction of aquaporin 1 but not aquaporin 4 messenger RNA in rat primary brain microvessel endothelial cells in culture.. J Neurochem..

[15] Haj-Yasein NN, Jensen V, Østby I, Omholt SW, Voiio J, Kila K (2012). Aquaporin-4 regulates extracellular space volume dynamics during high-frequency synaptic stimulation: a gene deletions study in mouse hippocampus.. Glia.

[16] Li X, Buxbaum JN (2011). Transthyretin and the brain re-visited: is neuronal synthesis of transthyretin protective in Alzheimer’s disease. Neurodegeneration..

